# Covid-19 Requires a Social Medicine Response

**DOI:** 10.3389/fsoc.2020.579991

**Published:** 2020-10-26

**Authors:** Lucas Jacob Trout, Arthur Kleinman

**Affiliations:** Department of Global Health and Social Medicine, Harvard Medical School, Harvard University, Boston, MA, United States

**Keywords:** social medicine, COVID-19, health equity, health disparities, global health

## Abstract

Covid-19 is an inherently social disease, with exposure, illness, care, and outcomes stratified along familiar social, economic, and racial lines. However, interventions from public health and clinical medicine have focused primarily on the scale-up of technical and biomedical solutions that fail to address the social contexts driving its distribution and burden. Fused with a moment of reckoning with racial injustice and economic inequality in the U.S. and across the world, these disparities charge policy leaders to develop, study, and share a response grounded in social medicine. As a yardstick for formulating, evaluating, and implementing health policy and care delivery, social medicine recommends at least three things: integrating health, social, and economic responses; bringing care to the points of greatest need; and focusing on broad equity-driven reforms in the pandemic's wake. With these tools, Covid-19 presents us with an opportunity to address the inequities that the disease highlights, exploits, and may otherwise entrench.

## Introduction

The informal charter of social medicine is generally taken to be a quote from physician-anthropologist Rudolf Virchow: “Medicine is a social science, and politics is nothing more than medicine on a large scale” (Ashton, 2006). There have been few moments in recent history when Virchow's words feel more instinctively relevant, or when we find them more clearly operationalized in acts of care from health workers, neighbors, and communities caring for those affected by Covid-19. From massive racial justice protests responding to the continued murder and oppression of Black Americans, to the tandem implementation of health and social interventions for the pandemic globally, we find broad resonance for the idea that health and social life are inherently connected—and that health care and social care work best delivered arm in arm (Baker et al., 2020; Cherry, 2020; Hardy and Logan, 2020).

Still, a far broader, more integrated, and institutionalized response will be required to redress the waves of suffering that have already arrived—and the many more still to come. Covid-19 charges communities and their health leaders to develop, study, and share a response grounded in social medicine: a rejoinder to this crisis that centers the experience and expertise of patients, families, health workers, and scientists engaging with the social realities of pandemic disease, and which cares for its medical, social, and economic dimensions at once.

Social medicine means understanding health and delivering care around an understanding of our deep sociality (Trout et al., 2018). It is a recognition that the lion's share of health determinants are social in nature, and that the highest-yield interventions are often social—in their design, implementation, targets, and outcomes—as well (Kasper et al., 2016). In this way, social medicine represents the practical application of a long scientific tradition that casts health and social inequities as twin phenomena, and which drives action to address each as contingent upon the other (Trout and Wexler, 2020). Grounding our local, national, and global pandemic responses in this framework will be one of the central challenges of our time.

## COVID-19 is a Social Disease

The initial framing of Covid-19—by journalists, celebrities, and even a few public health experts—invoked the notion of an “equal opportunity killer” (Blow, 2020). The dismantling of this narrative couldn't have come any sooner. It is clear that this disease, like nearly any other, seeks out and exploits the weak threads of our social fabric, stratifying exposure, illness, care, and outcomes along familiar social, economic, and racial lines (Van Dorn et al., 2020). Already well-documented are the staggering racial disparities in Covid-19 mortality in America, with emerging focus on the manner in which racism is driven into Black, Brown, and Indigenous bodies; similarly, across the world we have begun to map links between the severity of outbreaks and social and economic inequality (Blundell et al., 2020; Hardy and Logan, 2020; Kendi, 2020). As Americans, we find in our county not only an abdication of global leadership in providing care and aid, but also a mirror up to the lives we've collectively made: We find that it is the poor and marginalized who are at greatest risk of contracting Covid-19; it is the already-ill who weather the worst version of the disease; it is the uninsured who suffer at home for lack of access to care; and it is disproportionately the victims of these collective forms of structural violence who die (Chen et al., 2020; Williams and Cooper, 2020).

Covid-19 is a social disease. In the most straightforward sense, of course, infections move through social networks: that is, within families, through communities, and across networks of global commerce (Farmer et al., 2004). But the sociality of the disease runs deeper. The ability to social distance is socially-mediated, for example, by one's economic position, housing arrangement, employment, criminal record, and immigration status—all of which track race (meaning, racism) in America (Douglas et al., 2020). Public health recommendations assume access to rights and resources conferred only to some—for instance, running water, a utility still denied a full third of Navajo Nation homes (Krol, 2020). Information about the disease—true or false—moves through social and political networks, shaping a world of differential access to scientific knowledge and differential compliance with public health recommendations (Simonov et al., 2020). Finally, the allocation of scarce resources reflects the calculus of various social systems, from how families prioritize their needs to how protective equipment, diagnostics, therapeutics, and (someday) vaccines are distributed across localities, states, and nations (Emanuel et al., 2020; Van Bavel et al., 2020).

From a social medicine perspective, we already know—or can surmise—a great deal about Covid-19. Looking to historical analogs, we can anticipate many of the same reactions, from discrimination and blame to the reassessment of national and global priorities, as past plagues (Mann, 2020). We can expect efforts to de-socialize its meaning, causes, and care, for instance by falling back on tired stories of genetic predisposition, moral fault, and individual agency (Jones, 2009). By the same logic, we will likely find ourselves prone to overestimating the real-world efficacy of technological solutions such as vaccines and therapeutics, absent tandem efforts toward social care (Jones, 2020). Bearing witness to the differential impact of Covid-19 across populations, we can bank on conversations that center “risk factors” as a proxy for racial and class disparities, without adequate attention to the systems that structure, stratify, and sustain such risk (Farmer et al., 2006). Finally, we can expect fierce battles over the allocation of resources deemed too few to alleviate the physical, economic, and social suffering caused by the pandemic—and which, in both government and industry, the wealthy and white overwhelmingly control (Roberts and Mayo, 2019; Chayes, 2020; Congressional Research Service, 2020; United States Senate, 2020).

But Covid-19 is also a unique and historic event, striking just as the pervasive social, economic, and racial inequalities that define American life come clearest into view (Kendi, 2020). This pandemic represents a crisis of such exigence that we may be willing, as a nation and world, to entertain ideas about what a robust response might look like—and who is responsible for delivering the goods. Social medicine offers meaningful guidance in answering these questions.

## COVID-19 Requires a Social Medicine Response

Social diseases require social remedies. As a yardstick for formulating, evaluating, and implementing health policy and care delivery, social medicine recommends at least three things: integrating health, social, and economic responses; bringing care to the points of greatest need; and focusing on broad equity-driven reforms in the pandemic's wake.

### Integrating Health, Social, and Economic Policy Responses

Social medicine holds at its core a concept of health that unites the social, economic, and biological. Just as health is always a product of the confluence of these forces, care must be delivered through an understanding of their basic unity. This is not a novel perspective; the World Health Organization signified the relationship between these drivers of health—and the state's responsibility for their collective care—in its constitution more than 70 years ago (World Health Organization, 2020). The challenge, as always, is operationalization.

In the context of the Covid-19 pandemic, this means that we need to be clear-eyed about the purview of care required to address its health, social, and economic impacts. While the development of effective antivirals and vaccines is more than essential, other high-yield Covid-19 treatments will involve rapid, targeted, and sustained shifts in housing policy, unemployment and health insurance, jobs programs, sick leave, and monetary and fiscal policy. Such policies preferentially serve those at greatest risk for the worst outcomes from the disease itself, and from its economic and social fallout—but ultimately every person, family, and community stands to benefit greatly when the pandemic's spread and impact are lessened.

This is a time for investment in communities *as a health intervention*. Moreover, the best delivery platforms for these “social medicines” will almost certainly involve a level of horizontal integration rarely found in health care, government, education, and social institutions. Among other things, this means addressing the social determinants of health in clinical settings, as well as addressing the social arrangements governing the *distribution* of these health determinants on the policy level. It means bringing testing and care to accessible community settings, providing financial and logistical support to family caregivers, and facilitating the effective transfer of information across health and social sectors. Nations with the infrastructure and political will to implement and sustain such an integrated response provide us with early signals that wedding social care and health care is key to emerging from this pandemic not only healthier, but more economically and socially sound (Gentilini et al., 2020; Kolbert, 2020). The alternative—the economic decimation of the already-marginal, and consolidation of power among the protected and rich—is a path to greater health inequities, and greater social unrest, than America has yet seen (Davis, 2020).

### Bringing Care to the Points of Greatest Need

Social medicine means delivering care according to need, which, in the American situation, suggests a corrective and preferential option for the poor, marginalized, and those without ready access to care. A central challenge of the coming months and years will be to distribute public health, medical, social, and economic resources to the individuals, families, and communities in greatest need—with academic medicine, NGOs, and most of all governments playing essential roles. The historic and present-day underlayment of racism, economic and political exclusion, and other forms of structural violence against marginal groups in the U.S. suggests that this will, at best, be an uphill battle. But given the nature of infectious disease—where a single uncared-for case risks the health of all—this is a battle with our collective moral, economic, and physical lives at stake.

In the context of scarcity, the pandemic requires that we take stock of our collective resources and put them to use for the greatest possible good. This means the distribution of personal protective equipment (PPE) and deployment of clinicians to high-risk and highly-impacted communities. If and when a vaccine is proven safe and effective, the same principle should guide its distribution. It means appropriating funds for social programs that preferentially serve the poor, and for health programs that preferentially serve the already-sick. Finally, it means reimagining the act—and actors—of care itself: that is, providing training and financial support for family and community caregivers, reevaluating how we as a society care for health workers, and re-centering the moral dimensions of caregiving (and the moral lives of caregivers) who are dedicating—and in some cases sacrificing—their lives in acts of care.

### Focusing on Equity in the Pandemic's Wake

Pandemics inevitably remake societies. *How* precisely Covid-19 restructures American life is a question still very much unresolved—and the stakes could hardly be higher. Covid-19 presents us with an opportunity to correct the very inequities that the disease itself highlights, exploits, and may otherwise entrench. This is a pivotal moment in the American project: a chance to form that more perfect union, and to lay a new foundation where race, class, and social circumstance no longer define who lives, who suffers, and who dies.

Covid-19 hands us a veritable check-list of social problems to be solved. Immediately, the pandemic requires governments to ensure food on the table, roofs over heads, and quality, affordable health care for all. In the mid-term, it provides communities and their health leaders with opportunities to map and rank the social arrangements, laid especially bare by the pandemic, that both produce and are propagated by poor health. In the long-term, it presents us with the opportunity to reimagine these arrangements and to remake society in ways that center the just and humane treatment of all citizens, eradicate health disparities shaped by racism and class, and benefit the vast majority of Americans. Such reforms will undoubtedly make the next pandemic that much easier to weather: While Covid-19 is not an equal opportunity killer, there are very few winners when the global economy freezes, when political systems fracture, and when hundreds of thousands die. This is a moment to recognize that the health of one is tied to the health of all, and to take bold action to shape a more equitable future for our communities, nations, and world.

## Conclusions

Covid-19 is a social disease: a mass event of social suffering that requires an integrated, caring, and equity-focused response. Social medicine brings with it a set of tools for responding to the waves of suffering that have already arrived—in the form of disease and its attendant social fractures and economic wreckage—as well as others still to come. It provides a lexicon for the myriad ways that health tracks social and economic life, and a set of tools to undo these connections in favor of a fairer and more just society. It shapes the intellectual and moral scaffolding upon which our caregiving rests. Most of all, social medicine serves as a call to do something, now: to bring social care to our response, to care for those in greatest need, and to make equity central to our recovery. As health workers, policymakers, and citizens, this is our charge if we are to honor the trust of our communities in leading this response.

## Data Availability Statement

The original contributions presented in the study are included in the article/supplementary material, further inquiries can be directed to the corresponding author/s.

## Author Contributions

LT drafted the original manuscript in collaboration with AK, who contributed sections on racial disparities and theoretical discussions of social medicine. All authors contributed to the article and approved the submitted version.

## Conflict of Interest

The authors declare that the research was conducted in the absence of any commercial or financial relationships that could be construed as a potential conflict of interest.
